# Precision of patient specific screw holes locating surgical guide and pre-bent plates osteosynthesis versus classical workflow in management of class III mandibular fracture

**DOI:** 10.1186/s40902-025-00495-4

**Published:** 2025-12-19

**Authors:** Abdallah Gaber, Hussein Hatem, Mona el, Mohammed Omara

**Affiliations:** Cairo University, Cairo, Egypt

**Keywords:** Mandibular fractures, Class III, Computer-guided surgery, Open reduction and internal fixation

## Abstract

**Background:**

Several treatment modalities have been reported in the management of mandibular fractures using an alternative computer-guided approach through the utilization of different designs of guiding devices. However, these computer-guided methods do not always guarantee accurate anatomical bone reduction. This study aimed to assess the reduction precision of the computer-guided mandibular fracture and internal fixation using screw holes locating surgical guide, as presented earlier in the orthognathic surgery field in various studies to be applied in the field of mandibular traumatology, comparing it with the conventional approach.

**Methods:**

Twenty-six patients with ***Brown*** Class III mandibular fracture, defined by a single fracture line involving the body, parasymphysis or symphysis regions, were randomly assigned to two groups for open reduction and internal fixation. The study group underwent reduction and fixation using patient-specific screw-hole locating guide and pre-bent titanium miniplates, whereas the control group received conventional reduction and fixation with intraoperatively adapted titanium miniplates. Virtual reduction of the fractured mandible was performed in all cases of both groups utilizing CT scan and mimics software. Then, the actual postoperative mandibular model was superimposed over the virtually operated mandibular model based on predefined reference points and plans to obtain dental and bony linear measurements. The recorded measures were statistically analysed.

**Results:**

The actual postoperative mandibular model in the computer-guided group showed minimal deviation from the virtual mandibular model. While the deviation of the actual post operative model in the conventional group from the virtual model was higher, the difference in deviation between the two groups was statistically significant. The mean bony deviation was 0.09 ± 0.29 mm in the computer-guided group, versus 0.70 ± 0.33 mm in the control group *p* < 0.001. The mean dental deviation was 0.05 ± 0.16 mm in the computer-guided group versus 0.56 ± 0.32 mm in the control group *p* < 0.001.The mean operative time of the computer-guided group(1.49 ± 0.19)(hours) was significantly shorter than the mean operative time of control group (1.82 ± 0.37)(hours) which is statistically significant *p* < 0.001.

**Conclusions:**

The use of screw-hole locating guide and pre-bent plates enhanced surgical accuracy and efficiency. It also highlighted how patient-specific design can reduce dependence on surgeon experience and standardized outcomes in complex mandibular fractures.

**Trial registration:**

The study is registered at ClinicalTrials.gov Protocol Registration and Results System Receipt, ID: NCT05444829.

## Introduction

The mandible, the largest and only movable facial bone, is prone to fractures due to its anatomical and functional features [1]. ***Brown*** Class III mandibular fracture involving the body or symphysis require precise management. Open reduction and internal fixation (ORIF)are the gold standard for severely displaced fractures, ensuring proper alignment, stability, and function [2].

Conventional mandibular fracture management often struggles with limited intraoperative visualization of the lingual cortex, especially in complex sagittal fractures. This restricted view can compromise precise reduction, Poor reduction increases risks of complications such as malunion, malocclusion, and facial asymmetry, which may require secondary surgeries and increase patient morbidity [3, 4].

Recently, computer-guided technologies were utilized to ensure precise alignment of the bone fragments by means of bone borne surgical guide for ORIF of mandibular fractures. the application of screw holes locating guide and pre-bent plates osteosynthesis offered precise split segments alignment without the need for IMF and resulting in accurate postoperative occlusion in the field of orthognathic surgery [5].

To ensure more precise reduction and eliminate the need for intraoperative plate bending in the field of mandibular traumatology, this study introduced the use of pre-bent plates osteosynthesis, using screw holes locating surgical guide, in management of ***Brown*** Class III mandibular fracture. The aim was to assess the precision of patient-specific surgical guide and pre-bent plates osteosynthesis versus classical workflow in management of ***Brown*** class III mandibular fracture.

## Materials and methods

**Fig. 1:**
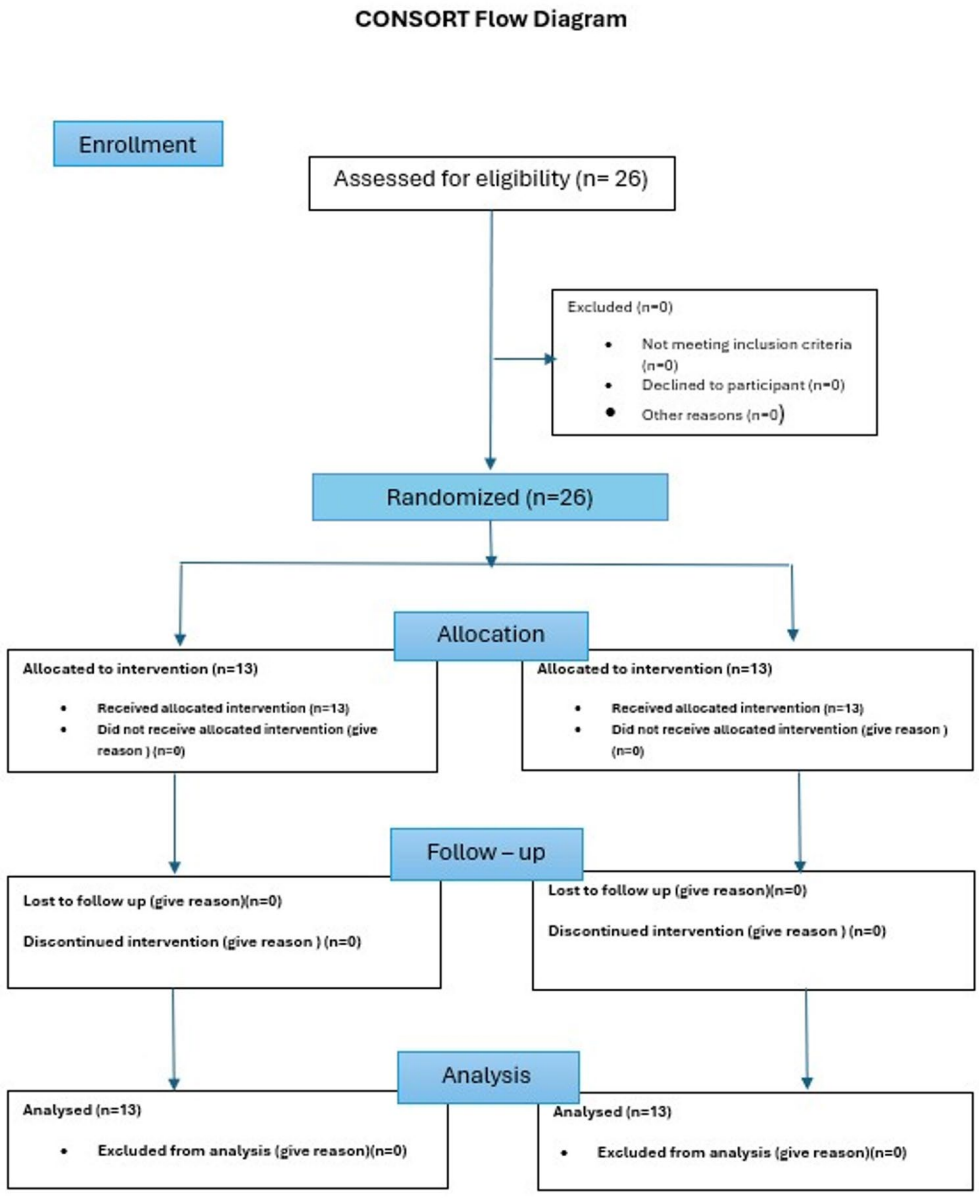
CONSORT flow diagram of participant recruitment and allocation. A total of 26 patients were assessed for eligibility. Patients with isolated mandibular class III fracture. Twenty-six participants were randomized equally into two groups: Group 1 comprised patients which underwent specific screw holes locating surgical guide and pre-bent plates osteosynthesis in management of class III mandibular fractures, while Group 2 comprised patients which underwent classical workflow in management of class III mandibular fractures. All participants received the allocated intervention and completed the study without loss to follow-up or exclusion from analysis

This randomized controlled trial included 26 patients consecutively enrolled from the Outpatient Clinic of the Department of Oral and Maxillofacial Surgery, Faculty of Dentistry, Cairo University. Ethical approval was obtained from the Ethics Committee of Cairo University (26/04/2022; No. 7422), and the study was registered at ClinicalTrials.gov (Receipt ID: NCT05444829). The trial was designed as a parallel, randomized, 1:1, double-blinded controlled study. The outcome assessor and participant were blinded. The methodology and reporting followed the Consolidated Standards of Reporting Trials (CONSORT) guidelines (Fig. 1).

Informed consent was obtained from all participants prior to enrollment. Patients were randomly assigned to two groups using a simple randomization method involving sealed, opaque envelopes (*n* = 13 per group). Each participant selected an envelope containing a group identifier (1 or 2). All 26 randomized patients were included in the final analysis, and no alterations to the study protocol were made after trial initiation.

### Sample size calculation

A power analysis was performed to achieve adequate statistical power for a two-tailed test of the null hypothesis, assuming no significant difference in reduction accuracy between the groups. Using an alpha level of 0.05, a beta of 0.2 (power = 80%), and an effect size (d) of 1.35 derived from a previous study [28], the calculated minimum sample size was 20 cases (10 per group). To account for potential attrition during follow-up, the sample size was increased by 25%, yielding a total of 26 patients (13 per group). The calculation was conducted using G*Power software version 3.1.9.7 [6]. Statistical analysis was performed with IBM^®^ SPSS^®^ Statistics Version 26 for Windows.

A thorough medical and dental history, followed by clinical examination, was carried out for all patients. Clinical measurements were taken to ensure patient adherence to the initial inclusion criteria prior to further investigations.

A preoperative digital panoramic radiograph with 1:1 magnification was taken for each patient as a primary survey to exclude the presence of any lesion at the area of interest or segment loss, followed by A pre-operative CT.

### Participant selection

#### Inclusion criteria

Patients with isolated ***Brown*** Class III mandibular fracture, defined by a single fracture line involving the body, parasymphysis or symphysis regions, indicated for open reduction and internal fixation (ORIF) was the main inclusion criteria for the current study, both male and female patients between 18 and 50 years of age, were eligible for inclusion.

#### Exclusion criteria

patients with additional facial fractures, patients with Pathological mandibular fractures associated with a lesion and the fully edentulous patients were excluded from the study.

## Preoperative Preparation

### In the control group

Dental impression was taken to fabricate dental cast. Dental cast was scanned to create 3D composite skull model. CT scans were obtained using multi-slice helical CT imaging machine(TOSHIBA Alexion multislice, 2011 Toshiba Medical Systems Corporation, USA). CT data were imported into Mimics 21.0 (Materialise N.V., Leuven, Belgium) for surgical planning. Bony structures were segmented and reconstructed in 3D. Virtual reduction was then simulated on the skull-dental model for future postoperative radiographic assessment.

### In the study group

**Fig. 2:**
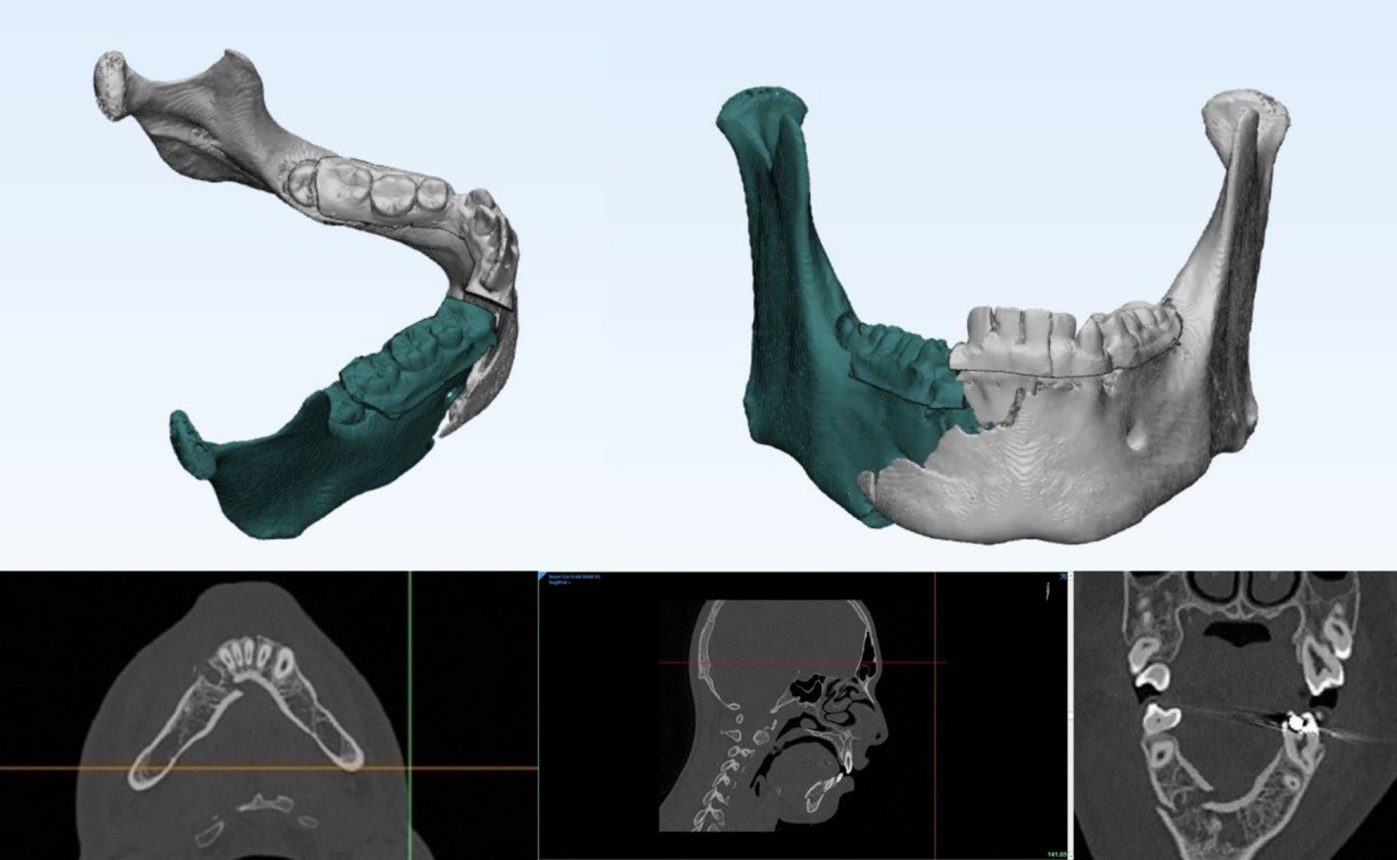
Snapshot of multiplanar Mimics (Materialise NV) screen showing the axial, coronal, sagittal and 3D of mandible fractur

**Fig. 3:**
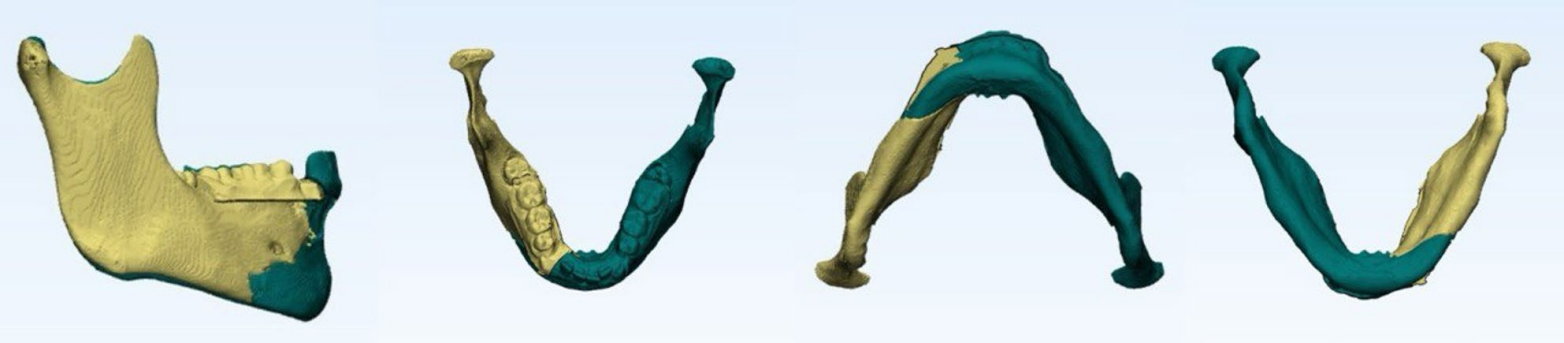
Snapshot of virtual reduction of mandible model

Dental impression was taken to fabricate dental cast. Dental cast was utilized for fabrication of a vacuum-formed stent then the dental cast was scanned to create 3D composite skull model. CT scans were obtained using multi-slice helical CT imaging machine(TOSHIBA Alexion multislice, 2011 Toshiba Medical Systems Corporation, USA) with patients wearing the fabricated vacuum-formed stent to maintain trauma in position during scanning. CT data were imported into Mimics 21.0 (Materialise N.V., Leuven, Belgium) for surgical planning(Fig. 2). Bony structures were segmented and reconstructed three dimensionally. Virtual reduction was then simulated on the skull-dental model to construct a corrected 3D mandibular model (Fig. 3).

The stereolithographic (STL) file of the corrected 3D mandibular model was exported to an additive CAM machine (Elegoo Neptune 4 Pro) and fabricated in white polyamide filament (PLA+) using fused deposition modeling (FDM) technology. Two titanium plates were selected (2.0 four holes spaced titanium miniplates) as tension band and (2.3 four holes spaced titanium plates) as compression band(Arab Engineers for Designs and Medical Instrumentation, Assiut, Egypt). The selected plates were bent and adapted in their position along the fracture line. After plates pre-bending, the plates were fixed in place using screws. Then the 3D model with plates fixed in place was laser scanned using a high-resolution 3D surface scanner, no opacifier material was utilized during scanning. The scanner used was a Medit i700 intraoral scanner (manufactured in South Korea by Medit Corp., headquartered in Seoul).

**Fig. 4:**
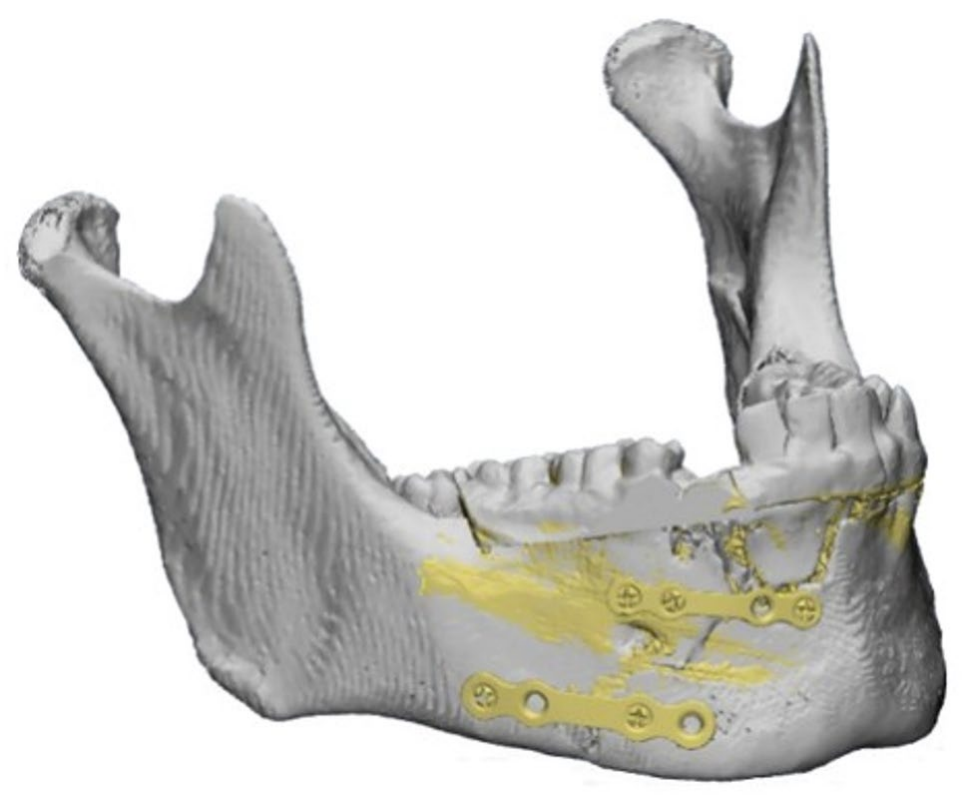
Snapshot of multiplanar Mimics (Materialise NV) screen showing the scanned mandible model with the pre-bent miniplates

**Fig. 5:**
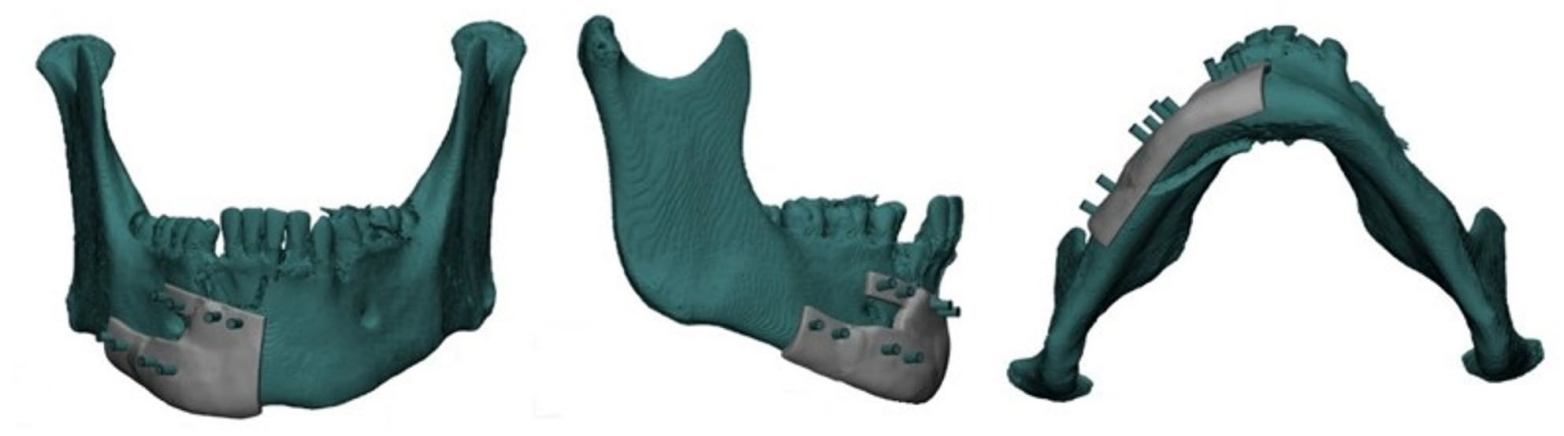
Snapshot of the multiplanar Mimics (Materialise NV) screen showing the designed screw holes locating guide adapted on the fractured mandible

The scanned mandibular model and pre-bent plates were then superimposed on the virtually reduced mandible (Fig. 4) to determine the exact screw hole locations on the fracture segments in their preoperative positions. Based on this alignment, a screw-hole locating surgical guide was fabricated to accurately transfer the virtual plan to the operative field (Fig. 5). The guide was designed to fit precisely on the bony surfaces of the trauma position, which had been maintained by the mentioned vacuum-formed stent placed previously over the teeth during the preoperative CT imaging. The screw-holes locating guide was produced using a resin 3D printer (Anycubic Photon M3 4 K that uses LCD masked stereolithography (MSLA) technology) and manufactured in white resin.

### Surgical procedure

In both groups, procedures were performed under general anesthesia with nasotracheal intubation, Articaine was injected into the labial sulcus along the incision line to provide hemostasis and control postoperative pain. The fracture in both groups was approached via an intraoral vestibular incision, ensuring a healthy cuff of tissue to facilitate wound closure and reduce the risk of dehiscence. The fracture was accessed, and the mental nerve was carefully dissected to prevent its injury.

### Control group

After achieving maximum intercuspation with maxillomandibular fixation, the fracture was reduced using a compression clamp. two types of plates were selected: tension band (2.0 four holes spaced titanium miniplates) and compression band (2.3 four holes spaced titanium plates) (Arab Engineers for Designs and Medical Instrumentation, Assiut, Egypt). The plates were intraoperatively contoured to the curvature of the mandible using bending instruments. The plates were placed along Champy’s ideal osteosynthesis lines According to the configuration of the fracture line,

### Study group

**Fig. 6:**
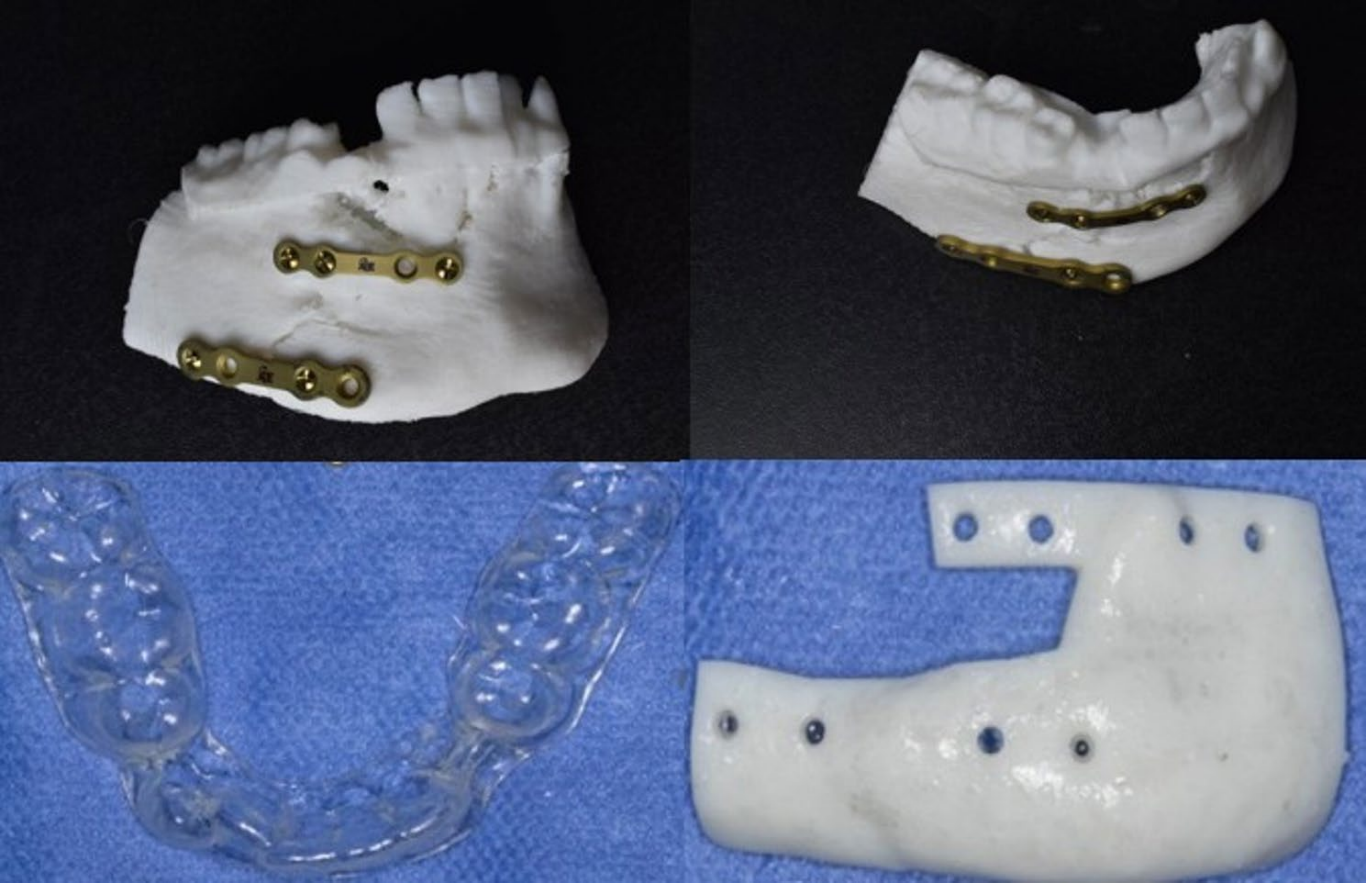
Intraoperative clinical photograph showing the execution tools for computer-guided reduced mandible: corrected 3Dmandible model, prebent titanium mini plates, and digitally fabricated surgical guide

**Fig. 7:**
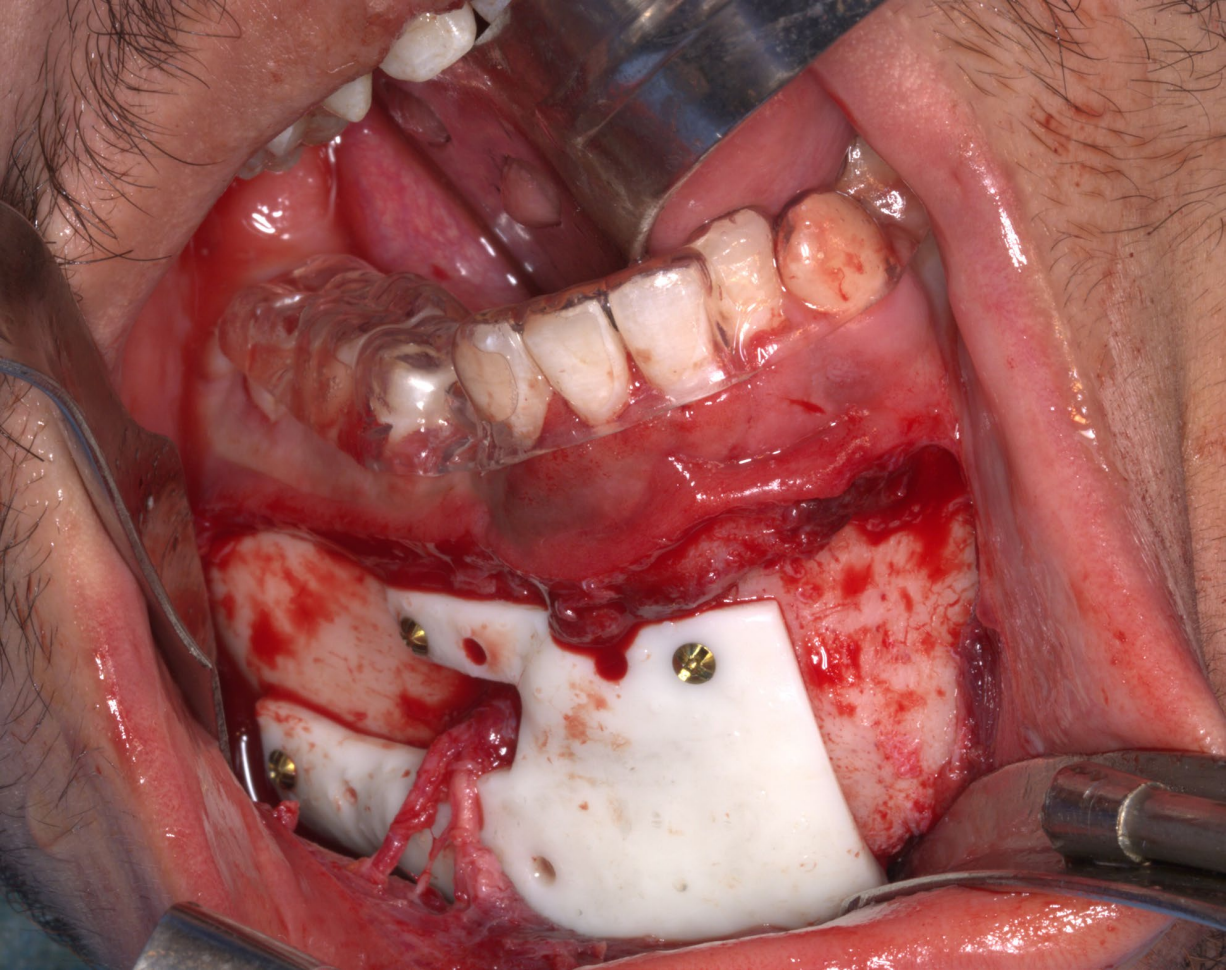
Intraoperative clinical photograph showing vacuum-formed stent to maintain trauma in position which acquired during preoperative CT scanning at which the screw holes locating guide was constructed in study group only

**Fig. 8:**
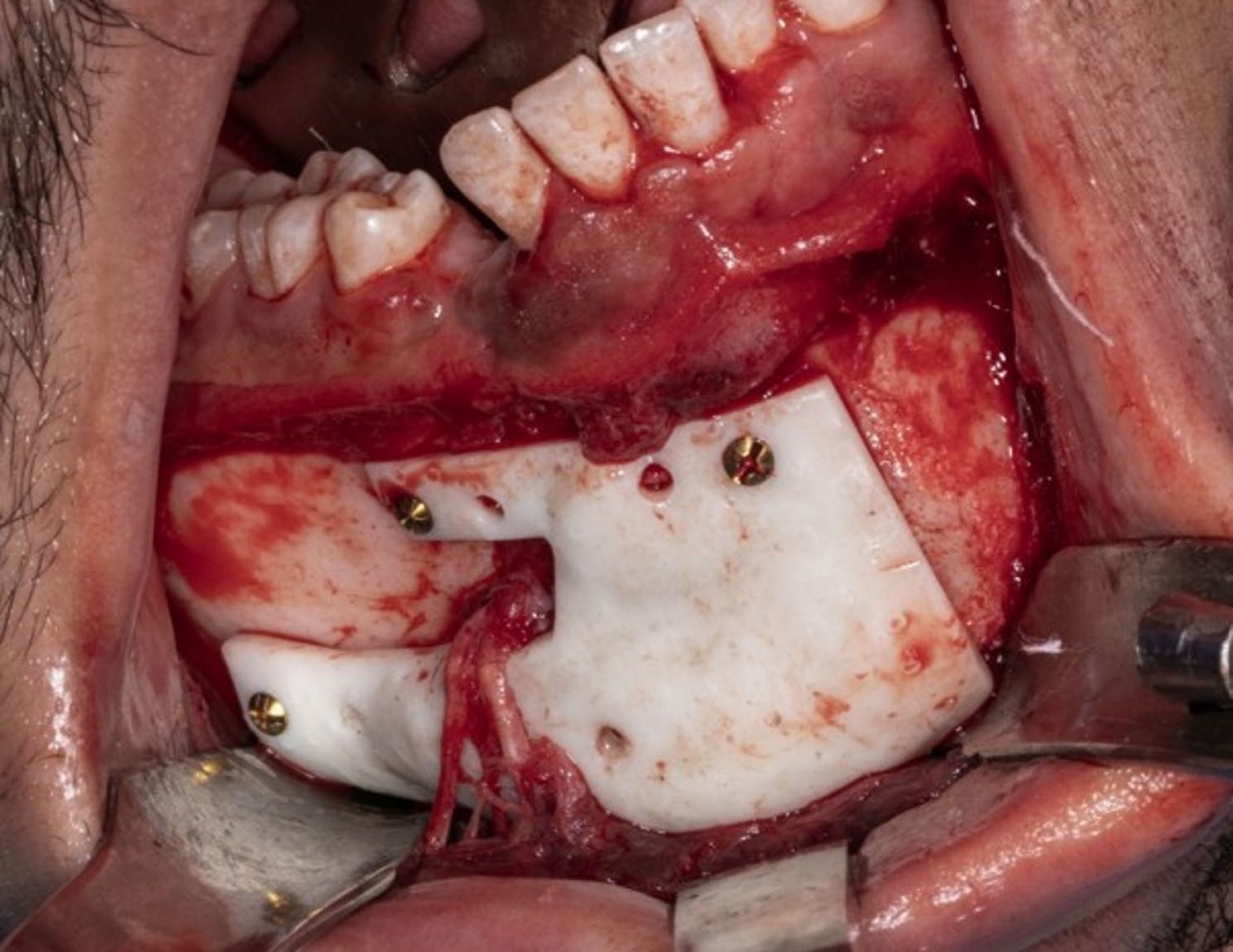
Intraoperative clinical photograph showing the screw holes locating guide anatomically adapted on the mandible

**Fig. 9:**
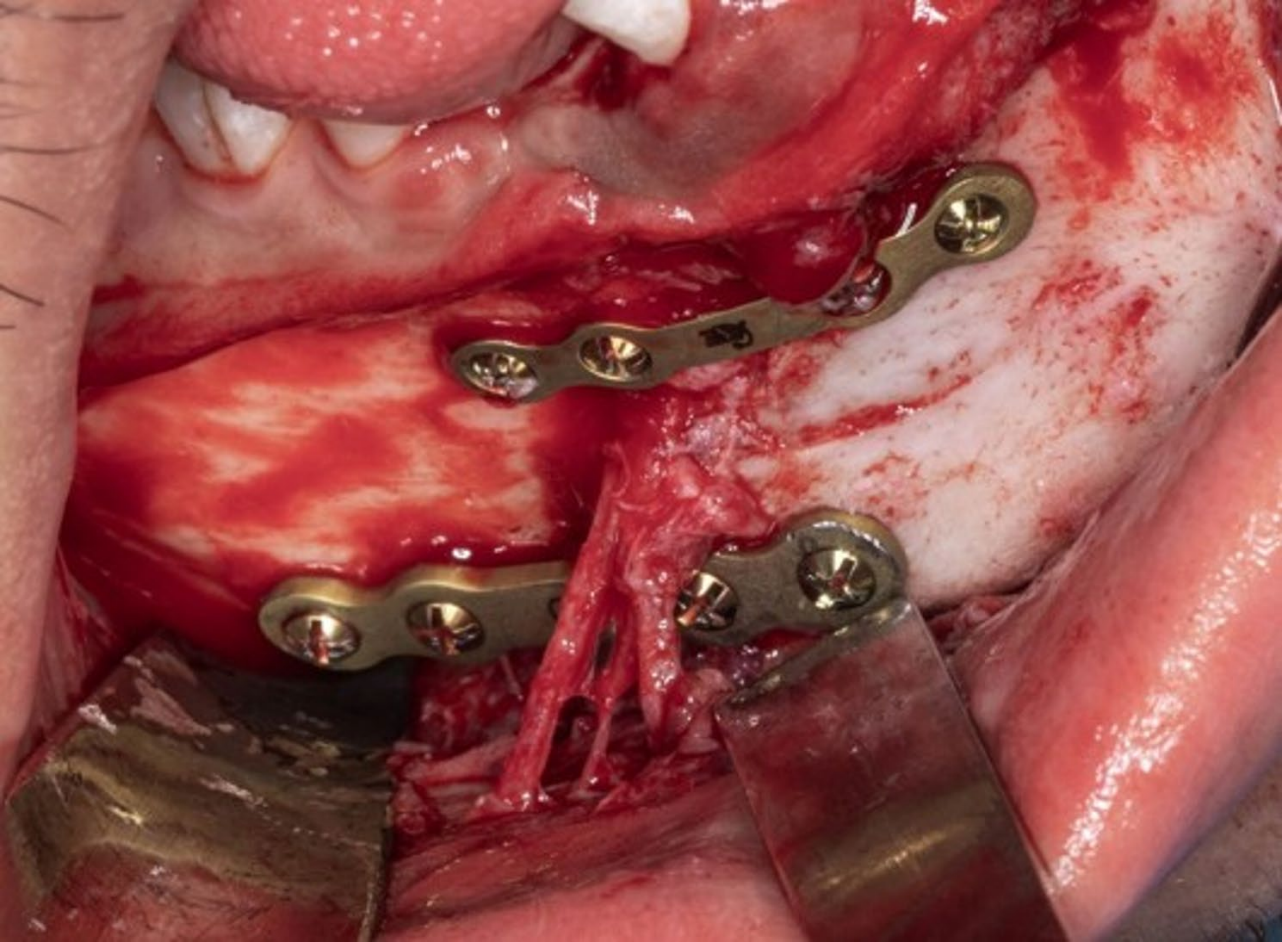
Intraoperative clinical photograph showing plate osteosynthesis

The prepared execution tools utilized were the pre-bent plates(Arab Engineers for Designs and Medical Instrumentation, Assiut, Egypt), screw-holes locating guide produced using a resin 3D printer (Anycubic Photon M3 4 K that uses LCD masked stereolithography (MSLA) technology) and manufactured in white resin and the previously mentioned vacuum-formed stent (Fig. 6). The fracture was approached through an intraoral vestibular incision leaving a healthy cuff of tissue along the gingiva to facilitate ease of closure and avoid dehiscence. The previously mentioned vacuum-formed stent utilized during CT imaging was placed intraoperatively over the teeth to maintain the position of the fractured segments over which the surgical guide was constructed during the virtual planning(Fig. 7). The screw-holes locating guide was anatomically seated on the fractured segments and fixed by mini screws(Fig. 8). The screw holes were drilled then the screw holes locating guide and the vacuum-formed stent had been removed. After the screw holes were drilled and the surgical guide was removed the fractured line edges were mobilized and cleaned from Any fibrous tissue entrapment. then the fractured segments were reassembled by the guidance of the pre-bent plates screwing(Fig. 9). The prebent plates were fixed in position utilizing screws of a predetermined lengths which were installed into the predetermined screw holes. plates fixation moved the fractured segments to the proper alignment without the need for IMF or bone holding forceps. Finally, all incisions were sutured with 4 − 0 resorbable polyglycolic acid(Vicryl 3 − 0 18” © Ethicon, INC 2005) sutures, which were performed in a continuous running fashion.

The time of the surgical procedures in both groups was calculated from beginning of the incision till completing of the suturing, the recorded data were sent for statistical analysis (Table 1).

**Table 1:** Intragroup comparisons and summary statistics for alignment time (hours)

Mean ± SD (hours)		*p*-value
Study	Control	
1.49 ± 0.19	1.82 ± 0.37	< 0.001*

### Postsurgical follow up and outcomes

**Fig. 10:**
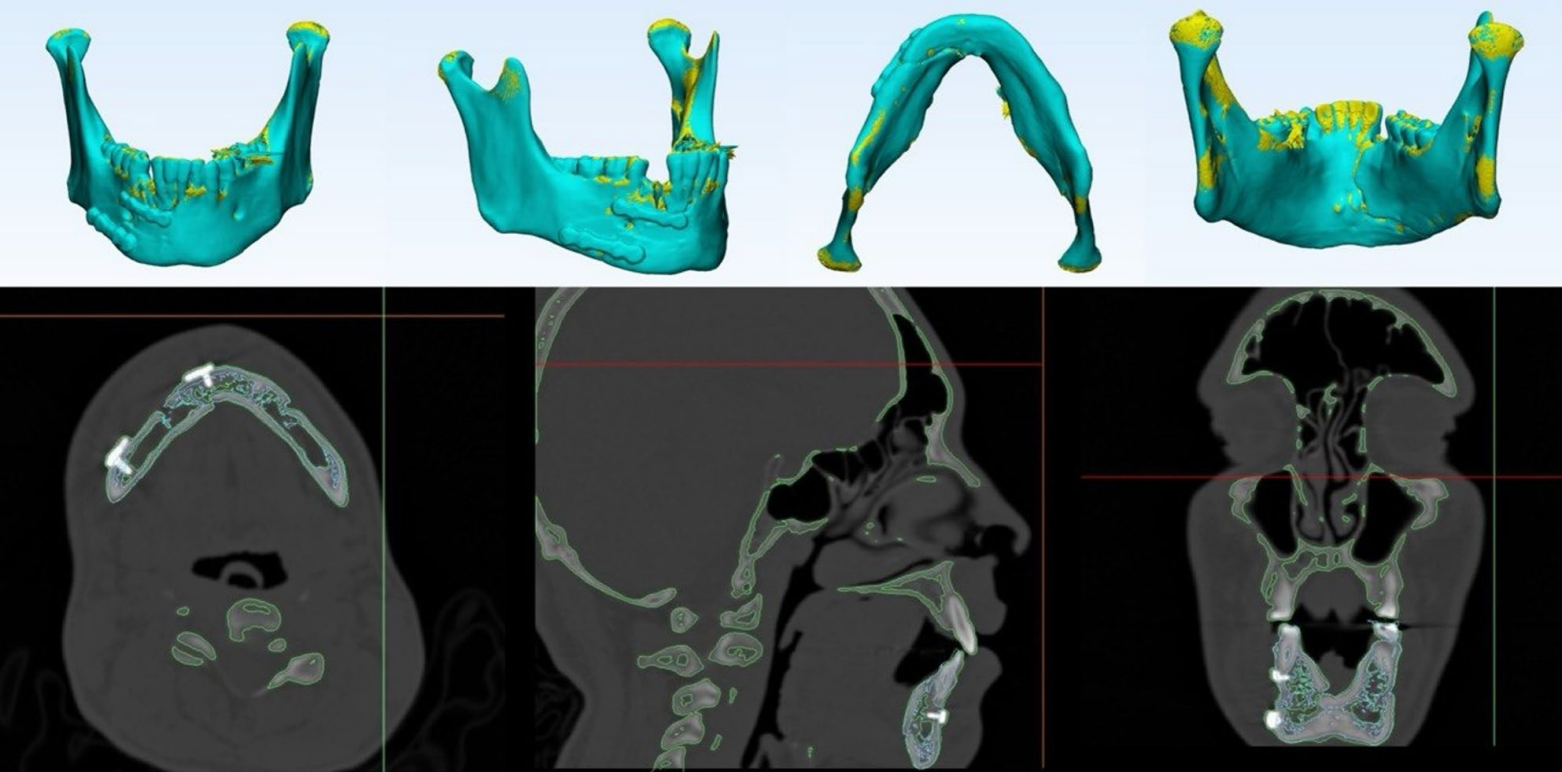
Snapshot of multiplanar Mimics (Materialise NV) screen showing the post operative radiographic assessment through super imposition of the actual post operative CT image over the virtual corrected CT image

Postoperative CT scans were obtained from all patient in both groups within the first week. they were analyzed using Mimics 21.0(Materialise N.V., Leuven, Belgium).For both groups, the actual postoperative mandibular model was superimposed over the virtually corrected mandibular model to determine any bony or dental deviations(Fig. 10).

Linear deviations were measured from dental landmarks(Mesioincisal Contact Points and Mesiolingual Cusp Tip) and bony landmarks (The tip of the coronoid, mental foramen, lingula, the Menton, pogonion and genial tubercles). These landmarks were measured Craniocaudally in relation to the Frankfort horizontal plane, anteroposteriorly in relation to the coronal plane and mediolaterally in relation to the sagittal plane using perpendicular lines to assess the surgical accuracy. All records were collected and submitted for statistical analysis.

Wound healing, dehiscence, malocclusion, and infection were assessed at the following time intervales: 1,2 and 4 weeks.

#### Statistical analysis

Categorical data (gender) were presented as frequency and percentage values and analyzed using Fisher’s exact test. Numerical data were expressed as mean and standard deviation (SD). Normality was assessed by inspecting data distributions (histograms and Q–Q plots) and confirmed with the Shapiro–Wilk test. Measurements and deviation data were nonparametric and analyzed using the Wilcoxon signed-rank test (for repeated measurements) and the Mann-Whitney U test (for independent measurements). P-values were adjusted for multiple comparisons using the False Discovery Rate (FDR) method. The significance level was set at *p* < 0.05 for all tests. Statistical analysis was performed with R statistical analysis software version 4.4.2 for Windows (R Core Team (2024). R: A language and environment for statistical computing. R Foundation for Statistical Computing, Vienna, Austria. URL https://www.R-project.org/.)

## Results

**Fig. 11:**
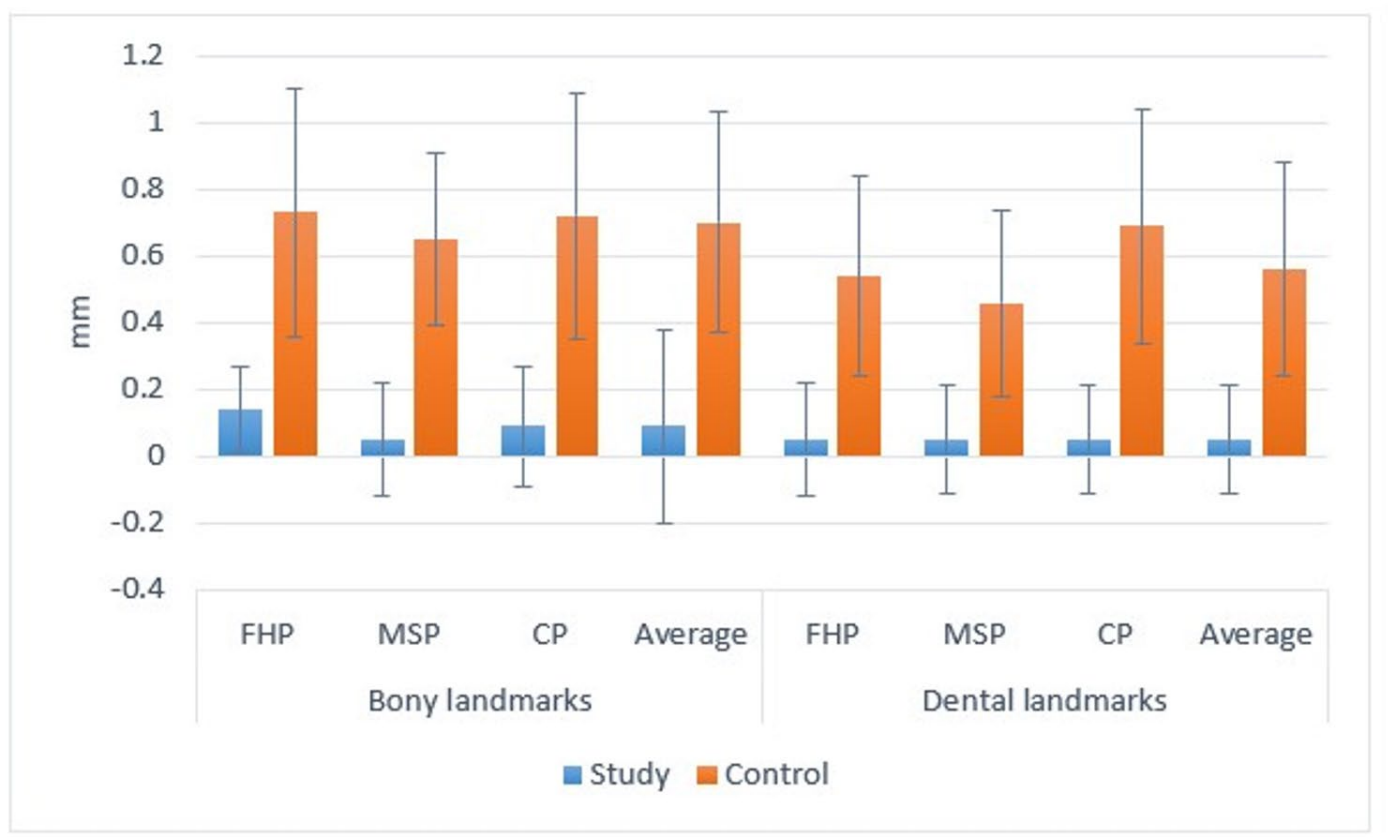
Bar chart showing mean and standard deviation for study group measurements

This clinical trial was conducted on 26 patients with isolated ***Brown*** Class III mandibular fracture (Fig. 11). Groups were statistically comparable regarding the gender (*p* = 1) and age (*p* = 0.977).

### Intergroup comparisons

#### Clinical outcomes

The average intraoperative time of the study group (1.49 ± 0.19) (hours) was shorter than that of the control group (1.82 ± 0.37) (hours) (*p* < 0.001). All patients in both groups demonstrated uneventful wound healing, except in 2 cases in the control group showed plate dehiscence at one week time interval, which was treated by warm saline irrigation during the follow-up period until complete healing. Complete healing was shown in the third week post operatively.

Regarding the malocclusion, all cases showed maximum intercuspation, except for two cases in the control group, which required heavy elastic application. The maximum intercuspation was obtained by the first month postoperatively.

#### Radiographic outcomes

The average bony deviation regarding Frank Horizontal Plane in the computer guided group was 0.14 ± 0.13, this deviation was statistically minimal compared to 0.73 ± 0.37 the control group and the difference between both groups was statistically significant p-value < 0.001. Moreover, the average dental deviation regarding Frank Horizontal Plane in the computer guided group was 0.05 ± 0.17, this deviation was statistically minimal compared to 0.54 ± 0.30 the control group and the difference between both groups was statistically significant p-value < 0.001.

The average bony deviation regarding mid sagittal Plane in the computer guided group was 0.05 ± 0.17, this deviation was statistically minimal compared to 0.65 ± 0.26 the control group and the difference between both groups was statistically significant p-value < 0.001. Moreover, the average dental deviation regarding mid sagittal in the computer guided group was 0.05 ± 0.16, this deviation was statistically minimal compared to 0.46 ± 0.28 the control group and the difference between both groups was statistically significant p-value < 0.001.

The average bony deviation regarding coronal Plane of the computer guided group was 0.09 ± 0.18, this deviation was statistically minimal compared to 0.72 ± 0.37 the control group and the difference between both groups was statistically significant p-value < 0.001. Moreover, the average dental deviation regarding coronal plane of the computer guided group was 0.05 ± 0.16, this deviation was statistically minimal compared to 0.69 ± 0.35 the control group and the difference between both groups was statistically significant p-value < 0.001.

The average bony deviation measured in the computer guided group was 0.09 ± 0.29 this deviation was statistically minimal compared to 0.70 ± 0.33 the control group and the difference between both groups was statistically significant p-value < 0.001. Moreover, the average dental deviation measured in the computer guided group was 0.05 ± 0.16 was statistically minimal compared to 0.56 ± 0.32 the control group and the difference between both groups was statistically significant p-value < 0.001. (Table 2)(Fig. 7).

**Table 2:** Intergroup comparisons and summary statistics for deviations

Landmark	Plane	Mean ± SD (mm)		p-value
		Study	Control	
Bony	FHP	0.14 ± 0.13	0.73 ± 0.37	< 0.001*
	MSP	0.05 ± 0.17	0.65 ± 0.26	< 0.001*
	CP	0.09 ± 0.18	0.72 ± 0.37	< 0.001*
	Average	0.09 ± 0.29	0.70 ± 0.33	< 0.001*
Dental	FHP	0.05 ± 0.17	0.54 ± 0.30	< 0.001*
	MSP	0.05 ± 0.16	0.46 ± 0.28	< 0.001*
	CP	0.05 ± 0.16	0.69 ± 0.35	< 0.001*
	Average	0.05 ± 0.16	0.56 ± 0.32	< 0.001*

Significant (p<0.05)

## Discussion

In craniomaxillofacial surgery, mandibular fractures represent a major proportion of trauma cases [6, 7], often requiring precise management to restore both function and aesthetics. Although treatment modalities are well established, ongoing limitations surround the conventional approach is recorded in the previous studies particularly in severely displaced bone segments [8, 9]. The emphasis on achieving precise reduction is essential for preventing malocclusion, nonunion, and overcoming the difficulties of intraoperative plates adaptation with reduction of intraoperative time and post-operative complications [10].

The integration of computer-guided techniques and CAD/CAM technology has revolutionized the management of mandibular fractures, offering remarkable enhancements in the precision of fracture reduction [11].

Various techniques have been developed for the treatment of mandibular fractures using several computer-guided approaches, which involve the utilization of different designs of guiding devices [12]. However, these computer-guided approaches do not always guarantee accurate anatomical bone reduction So, the author tried to cheek the accuracy of an orthognathic computer-guided approach and adopted it to be applied in the field of traumatology especially in the management of complex mandibular fractures. Based on the literature there are no studies trying to compare computer-guided approaches with conventional techniques. So, this study aimed to assess the accuracy of computer-guided reduction and fixation of ***Brown*** Class III mandibular fracture using the orthognathic protocol of screw-holes locating surgical guide and pre-bent plates osteosynthesis compared with the traditional technique, based on clinical and radiographic outcomes.

The present study focused on Class III mandibular fractures, which are characterized by a single fracture line affecting the body, parasymphysis or symphysis regions. The unfavorable configuration of ***Brown*** class III mandibular fracture, combined with muscular forces that displace the fractured segments, often induce difficulties during intraoperative fractured segments alignment and immobilization, to accurately restore the premorbid occlusion [13].

The current study utilized CT scans, which offer the advantage of higher image quality with improved contrast and resolution, which were acquired in a Digital Imaging and Communications in Medicine (DICOM) format, and imported into a surgical planning software Mimics 21.0 which has the ability to convert the DICOM files of the CT scan into 3D models by identifying and delineating the anatomic structures of interest in the CT scan through image segmentation.

Virtual visualization of the fractured mandible three dimensionally in conjunction with accurate segmentation method allowed for accurate fracture reduction with subsequent 3D printing of a corrected mandibular model over which the titanium plates were preoperatively adapted, bent, and fixed in its position. The pre-bent plates in its position were scanned and enter the virtual work up with its final pre-bent configuration and position for construction of its screw-holes locating guide.

This current computer guided approach was adopted in the current study group from the idea of reverse engineering technology applied in the orthognathic protocols, as the screw-holes and plate locating guide was constructed based on the scanned actual pre-bent plate in its actual postoperative position over the virtually corrected and printed 3D mandibular model [5, 14, 15].

The utilization of the vacuum-formed stent was to stabilize the fractured segment in position during preoperative CT acquisition and screw-holes surgical guide virtual designing and fabrication. This vacuum-formed stent was later utilized intraoperatively to reproduce and maintain the trauma position recorded during the preoperative CT scan, upon which the screw-holes locating guide was virtually constructed. The maintained fractured position aid by the vacuum-formed stent guaranteed the accurate seating of the screw-holes locating guide over the fractured mandible.

Regarding the flap design, the conventional technique in the current study typically involved narrow surgical access for the application of standard mini plates. While this approach reduced operative time and limits initial soft tissue manipulation, it often compromises local blood supply due to vessel compression induced by the tight flap’s retraction, contributing to increased postoperative edema with subsequent delayed wound healing and possibility of post-operative dehiscence and infection [16]. In contrast, the innovative use of screw-holes locating guide and pre-bent plates necessitates a wider surgical field to ensure accurate placement. Although this may initially seem more invasive, the broader exposure facilitates better visualization, more controlled soft tissue retraction, and preservation of the vascular network. Consequently, this technique is associated with improved perfusion, reduced postoperative edema, and potentially enhanced overall healing outcomes. These findings suggest that while the traditional method offers simplicity and speed, the computer-guided technique may yield superior biological and clinical benefits [17].

Regarding fractured segment alignment and intraoperative intermaxillary fixation (IMF), the current conventional approach utilizing arch bars or intermaxillary fixation screws to achieve accurate intraoperative occlusion, followed by fracture alignment with compression clamps and subsequent intraoperative plate adaptation and fixation—presented several challenges [18]. These intraoperative procedural steps are often labor-intensive, contribute to increased patient morbidity, risk of injury to dental roots, teeth avulsion, gingival lacerations and adjacent anatomical structures with subsequent longer recovery periods and potential occlusal discrepancies. Additionally, it may be less effective in patients with partial edentulism [19].

Regarding the intraoperative utilization of bone holding forceps in the conventional approach, their improper positioning, or its placement to clamp cortical bone of low-quality induced slippage, resulting in buccal cortical bone plate fracture during bone holding clamp clamp installment or additional trauma. Additionally, the use of bone holding forceps did not guarantee the exact lingual cortical alignment, which led to inaccuracies in reduction and suboptimal stabilization of the fractured segments [20].

On the other hand, the application of screw-holes locating guide and pre-bent plates osteosynthesis offered precise fractured segments alignment without the need for IMF or bone holding forceps application and resulting in accurate postoperative occlusion, which is reflected positively in the statistical analysis of the obtained radiographic data [21].

The accurate postoperative results observed in the study group were attributed to the precise positioning of the screw-holes and the use of anatomically pre-bent plates. These pre-bent plates facilitated accurate three-dimensional alignment of the fractured segments and maintained intraoperative anatomical relationships, including proper lingual positioning. Consequently, this ensured precise plates adaptation, minimized the risk of occlusal discrepancies, and improved the overall fixation accuracy.

Regarding the depth of the screws used for plates osteosynthesis, the computer-guided approach enabled precise preoperative determination of the optimal screw length, effectively minimizing the risk of inferior alveolar nerve injury. This level of preoperative planning enhanced both the safety and predictability of fixation. In contrast, the conventional approach relied solely on intraoperative estimation, which increased the likelihood of nerve injury or inadequate screw purchase, thereby compromising fixation stability.

The adopted computer-guided approach in the current study offered multiple clinical advantages over traditional freehand techniques. Notably, they reduced the risk of iatrogenic injury of the critical anatomical structures, including the inferior alveolar nerve and the dental apices. Moreover, they enhance the precision of plates placement, contributing to more predictable outcomes. In contrast to conventional methods, which are highly technique-sensitive and reliant on surgeon experience.

Regarding the reduction accuracy and the intraoperative time, the utilized computer-guided approach allowed for precise anatomical adaptation of the plates prior to surgery, thereby minimizing the need for intraoperative contouring. This coincides with the findings of a previous study conducted by ***Lethaus et al. (2012) and Goh et al.***. ***(2019***^***)***^ [22] ***.***They reported conclusively that pre-bending plates using 3D-printed anatomical models reduce intraoperative time and lead to superior conformity with the patient’s bone structure compared to intraoperative manual bending. Such preoperative adaptation contributes not only to enhanced fit but also to improve stability of the fixation, with a reduced incidence of screw misalignment, loosening and injury of underlying vital structures which are common complications that can compromise surgical outcomes in the conventional approaches [23–25].

Conversely, the traditional fixation technique in the current study required the surgeon to manually bend and adapt plates during the intraoperative workflow, which was a technique sensitive process [16]. The intraoperative manual bending increased the risk of inaccuracies in plate adaptation, which resulted in statistically suboptimal bone reduction. These factors elevated the likelihood of mechanical complications such as screw loosening, poor fracture stability, and infection due to stress-induced micromotion at the fixation sites which affect the clinical outcomes in the current conventional arm group. On the other hand, the computer-guided arm group showed favorable outcomes in the all-follow-up period. All patients of the computer guided arm group demonstrated uneventful healing, with no instances of wound dehiscence, plate exposure, or infection.

Postoperative assessments demonstrated the clear superiority of using patient-specific surgical guide for screw-holes localization and pre-bent plates osteosynthesis in the treatment of ***Brown*** Class III mandibular fracture. Across all selected bony and dental reference points, the dental deviations in the study group (0.05 ± 0.16 mm) were significantly lower than those in the control group (0.56 ± 0.32 mm) (*p* < 0.001). These deviations in both groups are negligible when compared to the clinically acceptable deviation range of 2 mm.

Although a 2 mm deviation is often considered acceptable, in clinical practice such a deviation may necessitate more than two weeks of postoperative rehabilitation using elastics to adjust occlusion. This can present a significant challenge in patient recovery. Moreover, minimal rotational discrepancies at the fracture site can propagate into substantial deviations in peripheral anatomical structures and occlusion, potentially affecting the overall treatment outcomes [26].

The computer-guided protocol by ***El-Gengehi et al.*** [27], focused solely on fractured segments immobilization. While the subsequent steps of plate adaptation and fixation were totally similar to the conventional workflow with MSP postoperative deviation range (SD = 0.39 mm) which is similar to the MSP deviation range of the conventional arm group in the current study (0.56 ± 0.32) (mm) and of inferior accuracy compared to our study arm group (0.05 ± 0.16) (mm). Similarly, the mean value of MSP deviation range in the study of ***Gaber et al.***, was high (2.4 mm) as they follow the same computer guided protocol that devoid the computer assisted plate pre-bending and fixation.

***Gaber et al.***,*** El-Gengehi et al.***,*** and Ramanathan M. et al.***, evaluated their results depending on solely bony landmarks on the other hand the current study employed dental landmarks to achieve more accurate postoperative measurements. This method enhanced the precision of spatial assessment when comparing actual surgical outcomes with planned virtual planning. In the analysis of the current study, the postoperative average distance of the dental reference points to the defined three planes (FHP, MSP and CP) was 33.90 ± 23.23 mm, which closely aligned with the corresponding measurements obtained from the virtual reduction model (33.85 ± 23.23 mm). The difference between these two values was not statistically significant (*p* = 0.530), indicating a high degree of accuracy in the virtual planning and execution process. These findings reinforce the validity of using of screw-holes locating guide and pre-bent plates osteosynthesis.

on the other hand, the postoperative average distance of the dental reference points to the defined three planes (FHP, MSP and CP) in the conventional arm group was 34.58 ± 24.23 mm, which varies from the measurements obtained from the virtual reduction model (34.12 ± 24.28 mm). The difference between these two values was statistically significant (p = < **0.001**) indicating that the conventional approach affects the precision of final post operative occlusion which was reflected negatively in the clinical outcome in some cases which required application of elastics two weeks postoperatively until establishment of postoperative stable occlusion.

Upon assessment of intraoperative surgical time, the operating time of the computer-guided approach was significantly reduced (1.49 ± 0.19) (hours) compared with (1.82 ± 0.37) (hours) in the conventional approach (*p* < 0.001) which is statistically significant, as the computer-guided approach facilitated fast and accurate 3D repositioning of fractured segments with precise prebend plates osteosynthesis. Similarly, in a recent systematic review, ***Chen et al.***., pointed out that patient-specific guides can significantly decrease orthognathic surgery time.

Finally, based on clinical and radiographic assessments, screw-holes locating surgical guide with pre-bent plates provided more precise management of ***Brown*** Class III mandibular fracture compared to traditional fracture reduction and fixation methods.

### Study limitations

This study requires a long-term follow-up for the participants to assess functional outcomes, patient satisfaction and post operative relapse.

## Conclusion

The adopted Computer-guided approach enhanced the precision and efficiency of ***Brown*** class III mandibular fracture reduction and fixation with significant reduction of the intraoperative time. The use of screw-hole locating guide and pre-bent plates enhanced surgical accuracy and efficiency. It also highlighted how patient-specific design can reduce dependence on surgeon experience and standardized outcomes in complex mandibular fractures.

## Data Availability

All data are available whenever requested.

## Abbreviations

CT: Computed Tomography
III: Three
ORIF: Open Reduction and Internal fixation
PRS: Protocol Registration and Results System
3D: Three-dimensional
FHP: * Frankfort horizontal plane*
MSP: Mid Sagittal Plane
CP: Coronal Plane
IMF: Inter-maxillary fixation
CAD/CAM: Computer aided design/computer aided manufacturing
DICOM: Digital Imaging and Communications in Medicine
SD: Standard Deviation
*CONSORT*: Consolidated Standard of Reporting Trials
STL: Stereolithographic
